# Do small RNAs unlock the below ground microbiome-plant interaction mystery?

**DOI:** 10.3389/fmolb.2022.1017392

**Published:** 2022-11-01

**Authors:** Roshan Regmi, C. Ryan Penton, Jonathan Anderson, Vadakattu V. S. R. Gupta

**Affiliations:** ^1^ CSIRO Microbiomes for One Systems Health, Waite Campus, Canberra, SA, Australia; ^2^ CSIRO Agriculture and Food, Waite Campus, Canberra, SA, Australia; ^3^ College of Integrative Sciences and Arts, Arizona State University, Mesa, AZ, United States; ^4^ Center for Fundamental and Applied Microbiomics, Biodesign Institute, Arizona State University, Tempe, AZ, United States; ^5^ CSIRO Agriculture and Food, Canberra, SA, Australia

**Keywords:** small RNAs, post gene regulations, microbiome, suppressive soil, RNA interference

## Abstract

Over the past few decades, regulatory RNAs, such as small RNAs (sRNAs), have received increasing attention in the context of host-microbe interactions due to their diverse roles in controlling various biological processes in eukaryotes. In addition, studies have identified an increasing number of sRNAs with novel functions across a wide range of bacteria. What is not well understood is why cells regulate gene expression through post-transcriptional mechanisms rather than at the initiation of transcription. The finding of a multitude of sRNAs and their identified associated targets has allowed further investigation into the role of sRNAs in mediating gene regulation. These foundational data allow for further development of hypotheses concerning how a precise control of gene activity is accomplished through the combination of transcriptional and post-transcriptional regulation. Recently, sRNAs have been reported to participate in interkingdom communication and signalling where sRNAs originating from one kingdom are able to target or control gene expression in another kingdom. For example, small RNAs of fungal pathogens that silence plant genes and vice-versa plant sRNAs that mediate bacterial gene expression. However, there is currently a lack of evidence regarding sRNA-based inter-kingdom signalling across more than two interacting organisms. A habitat that provides an excellent opportunity to investigate interconnectivity is the plant rhizosphere, a multifaceted ecosystem where plants and associated soil microbes are known to interact. In this paper, we discuss how the interconnectivity of bacteria, fungi, and plants within the rhizosphere may be mediated by bacterial sRNAs with a particular focus on disease suppressive and non-suppressive soils. We discuss the potential roles sRNAs may play in the below-ground world and identify potential areas of future research, particularly in reference to the regulation of plant immunity genes by bacterial and fungal communities in disease-suppressive and non-disease-suppressive soils.

## Introduction

The rhizosphere is comprised of soil closely associated with plant roots inhabited by a unique population of microorganism and plays a pivotal role in plant growth and health in both natural and managed ecosystems (Hartmann et al., 2008; Fierer, 2017). This region is a hotspot where interactions between plants and their associated microbes underpin the emergence of inter-kingdom collaboration that can benefit all the participants. Here, beneficial organisms across trophic levels interact with each other, the bulk soil microbiome and the host plant through signalling molecules and the provisioning of carbon and nutrients. Conversely, plant pathogenic microorganisms may also colonize the rhizosphere which can lead to a dysbiosis of the rhizosphere microbiome leading to a more disease susceptible host plant. The basis of these microbial interactions which can lead to positive or negative outcomes in terms of plant growth and health, involves both inter- and intra-kingdom cell-cell communication. The mediation of gene regulation by small RNAs (sRNA) has been found to control numerous biological processes in many diverse organisms (Finnegan and Matzke, 2003) such as fungal pathogens (Fulci and Macino, 2007; Zhou et al., 2012; Chen et al., 2014; Chen et al., 2015), plants (Yoo et al., 2004; Lelandais-Brière et al., 2010), humans (Gong et al., 2005) and bacteria (Hershberg et al., 2003; Vogel, 2009). Although bacteria lack the distinctive sRNA biogenesis pathways identified in eukaryotes, it is well established that control of target gene expression in bacteria by sRNA-like molecules is mechanistically similar (Liu and Camilli, 2010; Gottesman and Storz, 2011). A variety of sRNA-like molecules in bacteria have been shown to regulate cellular functions such as metabolism (Wassarman, 2007), virulence (Ramirez‐Peña et al., 2010), structure and stress response (Bessaiah et al., 2021) and biofilm formation (Taylor et al., 2017). Recently, sRNAs were reported to mediate interkingdom signalling in different organisms and pathosystems (Wang et al., 2017a; Teng et al., 2018; Zeng et al., 2019). For examples, plant-derived micro RNAs modulates the gut microbiome (Teng et al., 2018) and the virulence genes of plant pathogens (Wang et al., 2016) and fungal-derived microRNAs modulate the expression of plant immune genes (Wang et al., 2017a; Wang et al., 2017b).

Inspired by this evidence, we posit that sRNAs may serve as mediators for inter-kingdom communication between members of the rhizosphere microbiome and the host plant. However, it remains difficult to identify this process in the complex, dynamic and highly diverse rhizosphere environment where communication between members can occur multi-directionally (Mendes et al., 2013). Nevertheless, there are two areas of foundational knowledge that support our hypothesis. First, it is widely recognized that rhizosphere microorganisms are intimately involved in plant growth and immunity through their modulation of a variety of molecules and signals. Secondly, there is growing interest in the role of sRNAs as mediators in the regulation of functional and signalling pathways across different kingdoms, especially in regard to their ability to effectively migrate within or across microbe-plant cell boundaries (Huang et al., 2019). With the purification of extracellular vesicles (EV), it is now experimentally possible to parse mobile sRNAs from existing total sRNAs thus allowing better understanding of cell to cell interactions mediating inter-kingdom gene regulation (Cai et al., 2019; Zeng et al., 2019).

We hypothesize that further investigation into sRNAs, in combination with other techniques to untangle microbiome-plant interactions, will serve to better our understanding of the complex rhizosphere soil microbiome and the benefits it provides to plant health. To illustrate this, we discuss two distinct types of soil microbiomes originating from disease suppressive and non-suppressive soils. We propose three possible pathways of rhizosphere-mediated sRNA-based inter-kingdom signalling during the infection of wheat by the fungal pathogen *Rhizoctonia solani* AG-8. We also propose an integrated method to identify genes with key functions within the rhizosphere which includes traditional metagenomics and meta-transcriptomics approaches coupled with sRNA analyses. In addition, we include fungal and plant sRNAs as candidates for inter-kingdom signalling which are less explored components in soil-based disease suppression studies. An in-depth review of fungal and plant sRNAs was not included in this paper as they have been well-covered in previous publications (Ruiz-Ferrer and Voinnet, 2009; Billmyre et al., 2013; Wang et al., 2016; Singh et al., 2018).

## Mechanism of bacterial sRNA regulations

**FIGURE 1:**
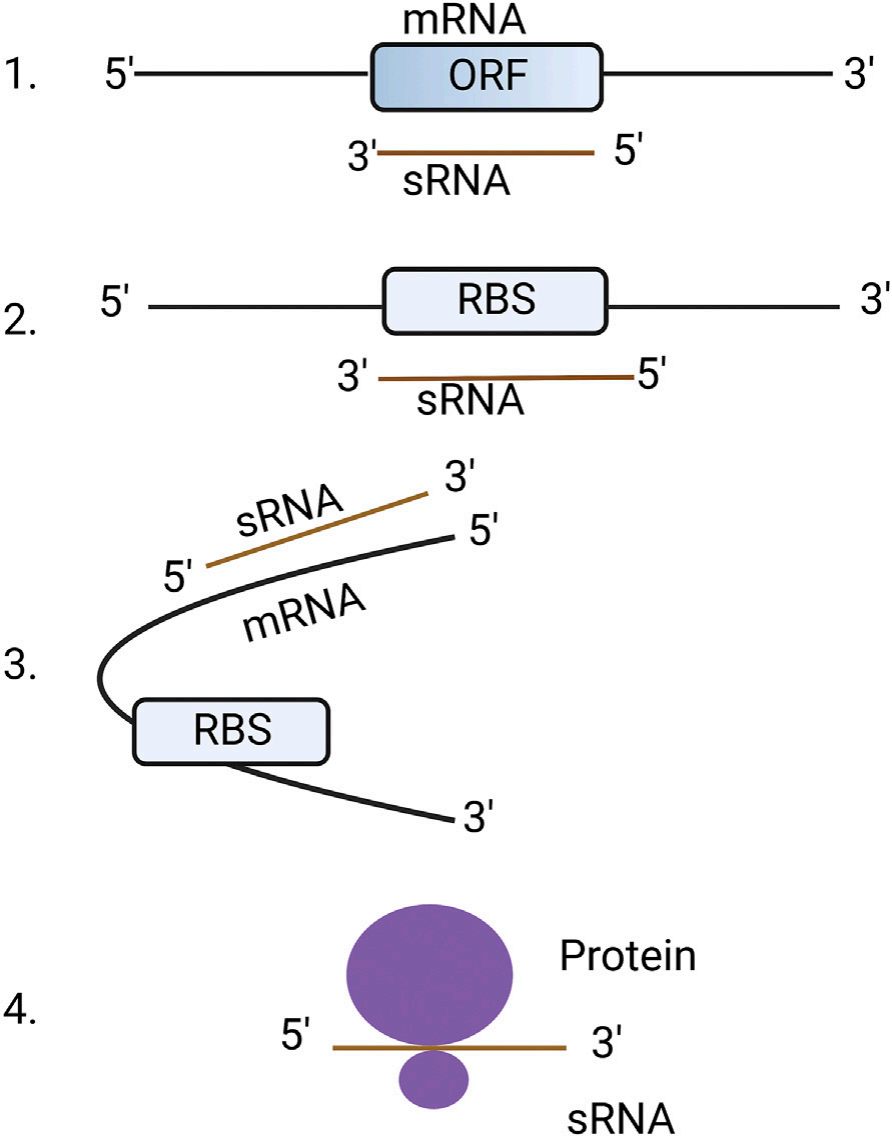
Mechanisms of bacterial sRNA regulation. 1. sRNA binds directly to open reading frame (ORF) of mRNA resulting in the degradation of RNA: RNA hybrid. 2. sRNA binds to ribosome binding site (RBS) resulting in blocking of translation. 3. sRNAs bind to the outside of the RBS causing a conformational change that allows or blocks access to the RBS. 4. sRNA directly binds to proteins to change their structure and function. The figure was produced with biorender.com (adapted from https://www.wikiwand.com/en/Bacterial_small_RNA).

Almost all eukaryotic and bacterial cells harbor two types of RNAs: coding and non-coding. Coding RNAs are those that are translated into protein (commonly called messenger RNAs or mRNAs) whereas non-coding RNAs are not translated into protein but otherwise regulate cellular functions (Hoe et al., 2013). Non-coding RNAs can be further characterized into different types based on their biogenesis and functions such as small non-messenger RNAs (snmRNAs), small non-coding RNAs (ncRNAs), untranslated RNAs (utRNAs), small RNAs (sRNAs) or non-protein coding RNAs (Brosius and Tiedge, 2004; Heidrich et al., 2006; Tjaden et al., 2006). For the purpose of this review, we used the term sRNAs to refer non-coding RNAs. Human, plant and fungal sRNAs are short non-coding RNAs between18-30 nt in length and regulate their target mRNAs through sequence complementarity. Bacterial sRNAs range from 50 to 300 nt but regulate the translation and stability of their mRNA targets in a similar way to higher eukaryotes (Wagner and Romby, 2015). Bacterial sRNAs can interact with mRNAs in four different ways, (i) by binding to the open reading frame (ORF) of mRNA causing degradation of RNA, (ii) by binding to the ribosome binding site (RBS) thereby blocking translation, (iii) by binding to mRNAs but not to RBS resulting in a conformational change which can enhance or supress translation, and (iv) by binding to protein targets directly, thus altering their functions (Waters and Storz, 2009) (Figure 1). An in-depth review concerning RNA-binding proteins that regulate the activity of bacterial sRNAs has been previously published (Quendera et al., 2020).

Based on the genomic loci of sRNAs and their corresponding mRNAs, they can be further classified into cis-encoded and trans-encoded. During cis encoding, both sRNAs and mRNAs are expressed from the same locus while in trans-coded sRNAs targets are expressed from different loci than sRNAs origin. Silencing by cis-encoded sRNAs of their targets might be achieved due to complementary sites and often a sole target for the given sRNAs while trans-encoded sRNAs often have multiple targets with partial sequence complementarity (Papenfort et al., 2010).

Though the discovery of the first bacterial sRNA 6sRNA was accomplished in 1967 (reviewed in (Wassarman et al., 1999)), its biological function was only identified in early 2000 (Wassarman and Storz, 2000). This sRNA was demonstrated to regulate RNA polymerase activity in a highly precise manner. In the early 2000s, the computational identification of bacterial sRNAs was accomplished, based on transcriptional signals and genomic features of known sRNAs encoding genes (Argaman et al., 2001; Rivas et al., 2001). With the more recent development of whole genome profiling and deep sequencing, it is now possible to directly detect sRNAs residing within the genome. For example, the complete genome sequence of *Escherichia coli* (Blattner et al., 1997) provided an opportunity to verify the presence of sRNAs which laid the foundation for future sRNA studies. Altogether, 24 sRNA-encoding genes were predicted of which 23 have been experimentally tested. Among these, 14 sRNA genes were novel (not discovered before) and exhibited abundant expression patterns under different physiological conditions (Argaman et al., 2001). Since then, several sRNA studies have been performed with *Salmonella enterica* (Pichon and Felden, 2005; Bohn et al., 2010; Chabelskaya et al., 2010; Eyraud et al., 2014) and *Staphylococcus aureus* (Pichon and Felden, 2005; Bohn et al., 2010; Chabelskaya et al., 2010; Eyraud et al., 2014) and varying cellular, metabolic, and biological process have been identified as regulated by sRNAs in different bacterial species. Bacterial chromosomes might harbour a few hundred to thousands of sRNAs with many yet to be discovered (Gottesman and Storz, 2011). Quite a number of computational tools have been developed to predict and validate sRNA:mRNA interactions including TargetRNA (Kery et al., 2014), sTarPicker (Ying et al., 2011), IntaRNA (Mann et al., 2017), and CopraRNA (Wright et al., 2014). Although these computational tools aid in the identification of sRNAs and their mRNA candidates, false positives or negatives hinder reliability. For example, while using these tools for the prediction of sRNAs from 18 enterobacterial species, CopraRNA was reported to have a low false positive rate in comparison to other tools (King et al., 2019). Likewise, in the fungal pathogen *Sclerotinia sclerotiorum* ten different computational tools predicted a varying number of miRNAs (Lee Marzano et al., 2018). Therefore, integrated experimental and computational approaches need to be combined to identify both sRNAs and their candidate target mRNAs*.*

Here, we discuss stress response and pathogenicity of bacteria which are two functions mediated by bacterial sRNAs relevant within the realm of rhizosphere communication and signalling. Briefly, bacteria have evolved with diverse cellular process to survive in fluctuating and stressful environments. A growing number of sRNAs have been reported to regulate stress responses in bacteria through fine tuning of signal transduction and regulatory proteins (González Plaza, 2020). Different studies have predicted and validated the involvement of a wide range of bacterial sRNAs that potentially have a role in regulation of responses to stresses such as variations in temperature, oxygen level and pH fluctuations in different species including *Staphylococcus aureus* (Abu-Qatouseh et al., 2010)*, Vibrio cholrea* (Raabe et al., 2011) and *Rhodobacter sphaeroides* (Adnan et al., 2015). In *R. sphaeroides,* a series of experiments revealed that SorY sRNA regulates the expression of takP mRNA which encodes a TRAP-T transporter. This sRNA/mRNA pair regulation has been shown to decrease the metabolite flux into the tricarboxylic acid cycle which is an adaptive response of bacteria during oxidative stress (Adnan et al., 2015).

Non-coding sRNAs are reported to regulate the virulence of various bacterial pathogens as diseases develop in humans (Toledo-Arana et al., 2007; Bordi et al., 2010; Bardill and Hammer, 2012) and plants (Liang et al., 2011). sRNAs can enhance the pathogenicity of bacteria by allowing them to adapt quickly to the environmental conditions of the host. They achieve this not with coarse “on or off” types of regulation but by regulating genes and transcription factors that fine tune the expression of target mRNA in response to local conditions (González Plaza, 2020). The role of sRNAs in bacterial virulence can be illustrated by their role in facilitating the carbon store regulator (CsrA) system (Bordi et al., 2010). CsrA is a sequence-specific binding protein used by bacteria for post-transcriptional regulation of gene expression. To do this, the CsrA protein binds to the 5’ untranslated end of early mRNA coding regions and inhibits translation thus altering mRNA turnover and/or transcript elongation. However, the amount of free CsrA protein is regulated by the relative level of the sRNA CsrB. When the CsrB sRNA is abundant, it competitively binds the CsrA protein preventing CsrA from interacting with target mRNAs which results in enhanced translation of the down-stream target gene mRNAs (Vakulskas et al., 2015). The synthesis and degradation of the CsrB sRNA are regulated in such a way that allows CsrA activity to be rapidly and efficiently adjusted in response to nutritional conditions and stresses (Romeo and Babitzke, 2018). In addition to their role in virulence, bacterial sRNAs are also involved in plant protections against pathogenic fungi. For example, in a beneficial rhizobacterial species *Pseudomonas fluorescens* three sRNAs were reported to protect cucumber from *Pythium ultimum* by regulating post-transcriptional derepress ion of biocontrol factors (Kay et al., 2005).

## The influence of the disease-suppressive rhizosphere microbiome in the rhizoctonia-wheat pathosystem

As a platform for grounding hypotheses concerning the role of sRNAs within the plant rhizosphere, we turn to a pathogen-soil microbiome interactive system; the fungal pathogen *Rhizoctonia solani* AG-8 and disease suppressive/non-suppressive soil microbiomes. Rhizoctonia root rot or bare patch disease is one of the most destructive soil-borne diseases resulting in significant losses in cereal crops in Australia, with an annual loss of $77 million (Murray and Brennan, 2010). Currently, no resistant varieties are available for wheat or barley and fungicide application and canola rotation are the only methods of control. Fungicide control is expensive and unreliable since incidence of infection and disease occur from early seedling phase to physiological maturity (Gupta 2022). Nevertheless, biological methods such as seed coating of hosts with antagonistic bacteria has been reported to decrease the impact of other root rot diseases including *Rhizoctonia cerealis*, and *Fusraium culmorum*. (Castro Tapia et al., 2020). However, following several years of no-till management practices, some soils have been shown to develop a disease suppressive state which minimises the expression of disease even though *R. solani* AG-8 is present in the soil (Wiseman et al., 1996; Davey et al., 2021). As an economical and low synthetic input method to control soilborne disease, understanding biological suppressive activities is key for sustainable agricultural production (Hayden et al., 2018). The disease suppressive activity is defined as the biological activity of resident microbial community which counteracts the pathogen and/or suppresses disease incidence or severity (Cook et al., 1995; Donn et al., 2014). Soils with suppressive activities against different soil-borne plant diseases have been identified across the globe (Anees et al., 2010; Schillinger and Paulitz, 2014; Schlatter et al., 2017). The relative abundance and diversity of microorganisms present in soils manifesting suppression of pathogenic plant activities has been shown through the use of DNA-based profiling both for bacteria (van Elsas et al., 2008) and fungi (Penton et al., 2014). Several bacterial species have been reported to contribute suppressive activities through various mechanisms such as antibiosis (an antagonistic association between two organisms, in which one is adversely affected) and plant growth promotion (Garbeva et al., 2004; Weller et al., 2007) while others were found to be abundant in disease suppressive soils in comparison to non-suppressive soils (Mendes et al., 2011; Donn et al., 2014). For example, *Proteobacteria, Pseudomonas,* and *nifH* harbouring bacteria such as *Burkholderia* (detailed in Table 1) possibly reflect “keystone species”. Despite the current evidence, the composition of key functional genes that govern suppressive activity and interconnectivity between soil-rhizosphere-plant microbiomes remains elusive. Although network analyses of differences in relative abundances of bacterial and fungal populations within suppressive and non-suppressive soils are useful to understanding connectivity within the microbial consortia, it is also important to understand the cause and effect of such interactions (Poudel et al., 2016). Currently, disease suppression research focuses on quantifying the microorganisms present in the soil microbiome. However, there remains knowledge gaps concerning how these suppressive communities are recruited and constructed under the influence of plant type and the identity of key functional traits that contribute to suppression. Here, we propose three cross-kingdom mechanisms of gene regulation mediated by sRNAs that potentially shape suppressive communities and demonstrate how these approaches can shed light on some of these fundamental questions.

**TABLE 1:** Bacteria identified in soil-borne disease suppression communities that have also been the subject of sRNA studies.

Bacterial population	Soilborne disease suppression references	Techniques used to study disease suppression	sRNA and gene expression related techniques	Related sRNA and gene expression studies	sRNA study references
*Paenibacillus*	Gupta (2014)	TRFLP, nifH amplicon sequencing	Transcriptomics	Transcriptomics profiling of carbohydrate utilization and nitrogen fixation	Sawhney et al., 2016; Shi et al., 2016; Brito et al., 2017
*Sinorhizobium*	Gupta (2014)	TRFLP, nifH amplicon sequencing	sRNA profiling	Role of sRNAs in nitrogen fixation	Del Val et al., 2007; Ulvé et al., 2007; Voss et al., 2009; Jiménez-Zurdo and Robledo, (2015); Baumgardt et al., 2016; Ceizel Borella et al., 2016
*Clostridium*	Gupta (2014)	TRFLP, nifH amplicon sequencing	sRNA profiling	Role of sRNAs in virulence	Romby et al., 2006; Michaux et al., 2014
*Bradyrhizobium*	Gupta (2014)	TRFLP, nifH amplicon sequencing	sRNA profiling	sRNA profiling in different *Bradyrhizobium* lineage	(Lelandais-Brière et al., 2010; Madhugiri et al., 2012
*Verrucomicrobia*e	Gupta (2014)	TRFLP, nifH amplicon sequencing	sRNA profiling	Role of faecal sRNAs in colorectal cancer	Tarallo et al. (2019)
*Burkholderia*	Mendes et al. (2011), Gupta (2014)	16S ribosomal DNA	sRNA profiling	Role of sRNAs in stress response and nutrient depletion	Khoo et al., 2012; Stubben et al., 2014; Mohd-Padil et al., 2017
		TRFLP, nifH amplicon sequencing			
*Pantoea agglomerans*	Gupta (2014)	TRFLP, nifH amplicon sequencing	sRNA profiling	Identification of Hfq-dependent (RNA chaperone) sRNAs in virulence	Panijel et al., 2013; Shin et al., 2019
*γ- Proteobacteria*	Mendes et al. (2011)	16S ribosomal DNA	Non-coding RNA profiling	Role of sRNAs in riboregulation	Jiménez-Zurdo et al., 2013; Becker et al., 2014)
*Firmicutes*	Mendes et al. (2011)	16S ribosomal DNA	sRNA + transcriptomics	mRNA degradation pathways in Gram-positive bacteria	Durand et al. (2015)
*Argobacterium tumefacians*	Gupta (2014)	TRFLP, nifH amplicon sequencing	sRNA profiling	Role of sRNAs in virulence	Wilms et al. (2012)
*Xanthomonas*	Mendes et al. (2011)	16S Ribosomal DNA	sRNA profiling	Role of sRNAs in virulence	Liang et al. (2011)
*Pseudomonas*	Mendes et al. (2011), Gupta (2014)	16S Ribosomal DNA	sRNA + transcriptomics + proteomics	Genome wide identification of non-coding RNAs, antisense activity, and new genes	Filiatrault et al. (2010)

## Plant host sRNAs role in bacterial and fungal infection

As analogous systems, at least at the coarse level, microbiomes of the human gut and plant rhizosphere may be influenced in similar fashions by sRNAs. Within the human gut, a balance between the host and the gut microbiome is essential to suppress the onset of disease. Defined sets of microbial signatures have been reported to be associated with human diseases (Tarallo et al., 2019; Wirbel et al., 2019). For example, specific bacterial taxa such as Clostridiaceae*, Ruminococcacceae,* and Fusobacteriaceae have been associated with colorectal cancer. Host derived sRNAs have been reported to act as important physiological regulators in human health (Tarallo et al., 2019). For examples, differential expression of faecal sRNAs have been shown to direct gut microbiome composition during colorectal cancer. In a similar fashion, plant sRNAs have been shown lately to regulate the gene regulation during the pathogen invasion. Moreover, plant hosts harbor the ability to influence both the structure and function of the associated, interacting rhizosphere microbiome (Katiyar-Agarwal and Jin, 2010). More concisely, in the context of plant diseases, sRNAs are reported to regulate gene expression within both the host and fungal pathogen (Fujii et al., 2005; Li et al., 2010; Xia et al., 2020; Regmi et al., 2021) and can silence fungal virulence genes through the secretion of EV particles (Cai et al., 2018). EVs are lipid-bound secretion of cells into the extracellular space that comprises lipids, nucleic acids, and proteins (Zaborowski et al., 2015). Recently, these particles are reported to be a carrier of sRNAs across the different cells (Zhang et al., 2020). In wheat, host induced silencing of pathogen (*Blumeria graminis* f. sp. tritici) genes by wheat sRNAs enhances quantitative plant resistance (Schaefer et al., 2020). Furthermore, wheat was shown to use sRNAs to regulate its own endogenous defence genes in response to the fungal pathogen *Zymoseptoria tritici* (Ma et al., 2020). Likewise, a novel canola sRNA also mediates defence-related ethylene response factor genes under infection by *S. sclerotiorum* (Regmi et al., 2021). Although there is no direct evidence of plant sRNAs mediating bacterial gene activity, as has been demonstrated for fungi, plant sRNAs contributes to antibacterial resistance by repressing signalling pathways (Navarro et al., 2006; Zhang et al., 2011). It has been shown for the first time that miR393 from Arabidopsis was induced upon the infection of *Pseudomonas syringae* and repressed auxin signalling pathways which restrict *P. syringae* growth (Navarro et al., 2006). In another study, non-pathogenic, virulent and avirulent strains of *Pseudomonas syringae pv. tomato* in Arabidopsis results in differential expression of 15, 27 and 20 miRNA families that regulate plant hormone and signalling pathways (Zhang et al., 2011). This suggests that plants can reprogram their transcriptional response to protect themselves against bacterial infection through the use of sRNAs.

This opens up new questions which current technology can serve to answer: To what degree do plant sRNAs have a role in shaping the structure of a disease suppressive microbiome? What types of microbial regulatory genes/transcription factors are regulated by plant sRNAs? It has been reported that plants can send sRNAs through EVs to invading fungal tissues in order to silence fungal virulence genes (Cai et al., 2018), however any role of plant derived EVs in shaping the rhizosphere microbiome towards a disease suppressive state is not known.

## Fungal sRNAs role in the regulation of plant mRNAs

An involvement of fungal sRNAs in the pathogenicity of different plant pathogenic fungi such as *Phytophthora* (Qiao et al., 2015), *Magnaporthe oryzae* (Raman et al., 2017), *Botrytis cinerea* (Wang et al., 2017a), and *Sclerotinia sclerotiorum* (Derbyshire et al., 2019) has been described. These fungal derived sRNAs regulate functions including effector gene regulation, transposable elements regulation, stress response, appressoria formation, sclerotia development and suppression of host immunity. In wheat, several studies have reported cross-kingdom silencing of plant genes by fungal sRNAs. For example, *Puccinia striiformis* and *Fusarium graminearum* sRNAs silence pathogenesis-related 2 (PR2) and resistance-related target genes (Chitin elicitor binding protein) (Jian and Liang, 2019), respectively, resulting in the suppression of wheat defence mechanisms (Wang et al., 2017b). Similar inter-kingdom signalling was reported in other pathosystems including in the *Botrytis cinerea* and Arabidopsis pathosystem, where *B. cinerea* sRNAs silence plant immune related genes in the plant host Arabidopsis (Wang et al., 2017a), and the Fusarium-wheat pathosystem where fungal sRNAs suppress the activity of wheat mRNAs (Jian and Liang, 2019). In contrast, in the Zymoseptoria-wheat pathosystem strong evidence for cross-kingdom RNAi was not found, suggesting this phenomenon might not be universal (Kettles et al., 2019). In addition to plant-pathogen interactions, plant-symbiont interactions have also been shown to involve interkingdom sRNA signalling (Silvestri et al., 2019; Silvestri et al., 2020). For example, sRNAs derived from two AMF fungi, *Gigaspora margarita* and *Rhizophagus irregularis,* were shown to target 11 common genes in the host *Medicago truncatula,* suggesting sRNAs play a central role in conserved strategies to condition the host plant for colonisation by the symbiont. Given the evidence for interactions between soil-borne fungi and plants at the sRNA level, we can ask whether Rhizoctonia sRNAs regulate plant immunity genes and whether this cross-kingdom gene regulation is altered under suppressive and non-suppressive soil conditions.

## Bacterial sRNAs mediate the silencing of plant mRNAs

Bacterial sRNAs of 50–100 nt regulate the expression of protein coding host RNAs through imperfect base pairing of short regions (15–20 nt) (Altuvia, 2007). Such regulation might be achieved by either degradation of the resulting double stranded RNA or by blocking translation through interference with the ribosome binding site. High throughput sRNA sequencing of phytopathogenic bacterial species has revealed putative virulence-related sRNA candidates (Wiedenheft et al., 2012; Wilms et al., 2012). For example, in *Agrobacterium* species, 228 sRNAs were predicted from four replicons among which 20 were experimentally validated with RNA gel blot analysis. One 113 nt sRNA encoded in the intergenic region of the Ti- plasmid was expressed abundantly during virulence and potentially regulated by the VirA/VirG system (Wilms et al., 2012). VirA/VirG is a two-component system needed to induce virulence gene expression in *Agrobacterium sp.* and is induced by various plant signals, including acetosyringone (Stachel and Nester, 1986). It is worth mentioning that *Agrobacterium* species in the soil microbiome facilitate interkingdom gene transfer between bacteria and plants, and as such, it will not be surprising if silencing of plant genes by *Agrobacterium* sRNAs is also found to occur. However, the plant microRNA pathway is essential for *Agrobacterium* disease development (Dunoyer et al., 2006) suggesting a role for RNA silencing in susceptibility to *Agrobacterium* sp. A growing body of evidence also suggests that non-coding bacterial RNAs have evolved ways of evading host defence mechanisms (Zur Bruegge et al., 2017). A 115 nt long *Xanthomonas* sRNA was reported to regulate 63 genes related to signal transduction, transcriptional, post-transcriptional and virulence functions in pepper plants. (Schmidtke et al., 2013). Recently, it has also been shown that rhizobacterial sRNAs silence soybean genes involved in root/hair development which would affect rhizobial infection and nodulation formation (Ren et al., 2019). These studies suggest that bacterial sRNAs present in the rhizosphere may cross to plant tissues and regulate host genes. However, how this transfer to plant tissue occurs remains unknown. From the perspective of our disease suppression model, it would be interesting to compare the expression of plant immunity genes in the two different soils in concert with sRNA profiling. However, it would be prudent to characterize the microbiome composition and function beforehand. One approach would be to first compile reference-like genomes of the bacterial species in suppressive communities to determine the source of the sRNAs and then to map these back to plant transcriptomic data to identify any cleavage events. If disease suppression is driven by bacterial sRNAs then we would expect the cleavage of plant immunity genes in suppressive soils to be lower than in the non-suppressive soils.

Although bacterial and fungal interactions are enormously important in the agricultural, environmental and medical sectors, the specific molecular mechanisms that underlie their interactions remains largely unknown. A recent study provides some insight by providing biomolecular evidence that the bacteria *Pseudomonas picium* modified histones of the wheat pathogenic fungus *Fusarium graminearum* and reduced its virulence due to the impact of phenazine-1-carboxamide (Chen et al., 2018). However, there is currently a lack of evidence concerning bacterial sRNAs based mediation on the silencing of fungal sRNAs though, theoretically these interactions would be expected to occur. However, the mechanisms and delivery method for bacterial derived sRNAs to fungal pathogens remains unclear. At this stage, it can only be hypothesized that after the generation and maturation of bacterial sRNAs they might be transported through EVs (extracellular vesicles), bound to *Argonaute* proteins of fungal cells or move out freely (Mendes et al., 2013). Recent studies on -omics tools suggest the involvement of several bacterial groups and some fungal genera in disease suppressive activity to *R. solani* AG8 in crops like wheat (Penton et al., 2014; Davey et al., 2021). Whether sRNAs act as a mediator of gene regulation between these bacterial and fungal communities is uncertain and deserves further investigation. For example, it would be interesting to determine whether regulation of fungal virulence genes is mediated by bacterial sRNAs.

**FIGURE 2:**
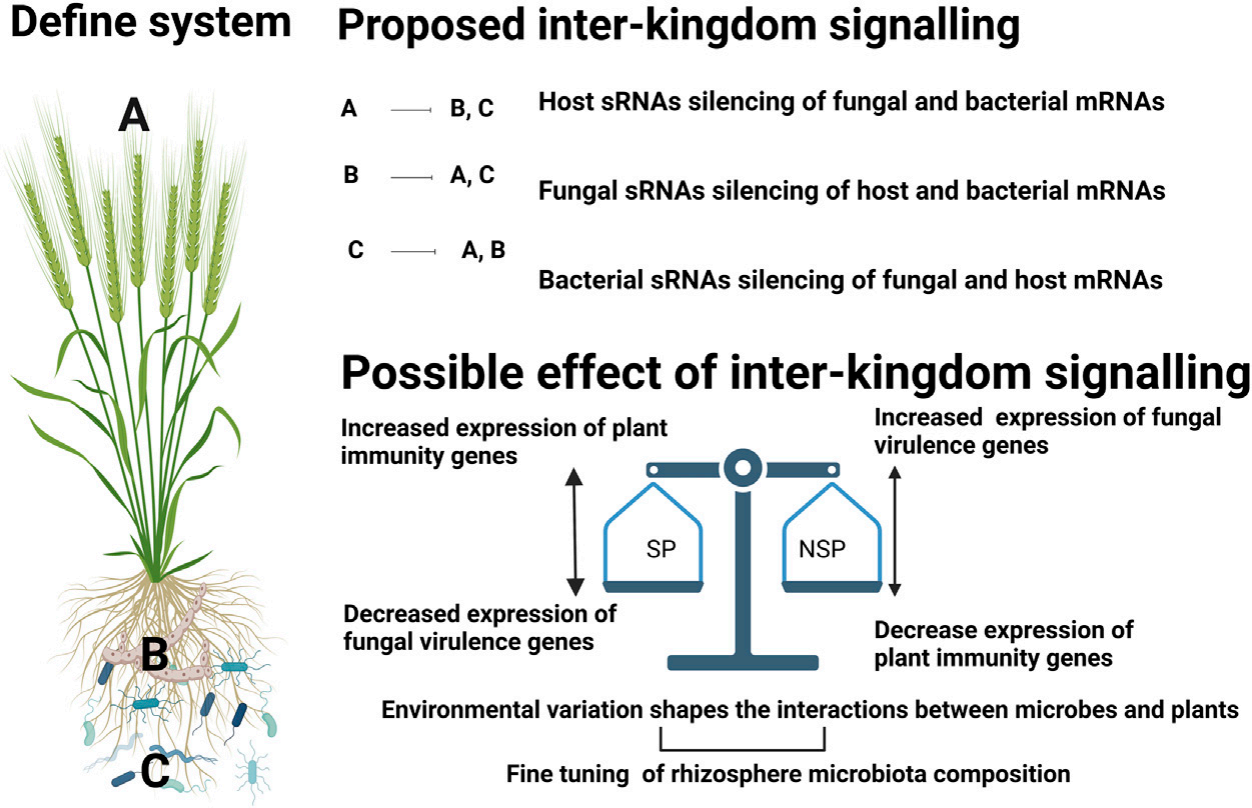
Schematic for possible ways of sRNA mediated gene regulation in wheat-rhizoctonia-microbiome interactions and possible effect of such interactions in the context of disease suppressive soils. We proposed involvement of sRNAs from three components: i) plant host, ii). fungal pathogen, iii). bacterial community, with possible multidirectional interactions. During the complex interaction of plant association with rhizosphere community, Inter-kingdom signalling might happen in such a way that plant sRNAs **(A)** could be taken up by fungal **(B)** (Cai et al., 2018) and bacterial **(C)** cells thereby mediating regulation of their genes (Teng et al., 2018). In the second case, fungal pathogens can secrete sRNA effectors into host cells to suppress host immunity (Wang et al., 2017a) and bacterial transcripts. Although there is no direct evidence of fungal sRNAs silencing bacterial genes, this concept may bear testing in suppressive soils as members of the bacterial community have been identified as key components that contribute to the suppressive capacity of a soil. In the third case, bacteria can incorporate their sRNAs to modulate host genes (Ren et al., 2019) and also can alter the expression of fungal genes (Chen et al., 2018). The possible effect of this inter-kingdom signalling in suppressive (SP) and non-suppressive (NSP) soils could be as follows: 1. In SP; either bacterial or host sRNAs can decrease the expression of the fungal virulence genes resulting in increased expression of plant immunity genes. In NSP soils the fungal cell may be free to secrete sRNA virulence factors that supress expression of plant immunity (such as disease resistance proteins, WRKY transcription factors, s AP2/ERF factors as evident from previous studies (Wang et al., 2017a; Wang et al., 2017b; Jian and Liang, 2019)) thereby increasing the disease symptoms. Although it is out of the scope of this paper, it is important to consider intra-kingdom signalling within **(A–C)** that might play a role in fine tuning the composition and/or function of the microbiome along as well as respond to environmental factors.

Figure 2 outlines the hypothesis concerning possible cross-kingdom silencing and the possible cause and effect interactions that occur in suppressive and non-suppressive soils. To be able to identify and design efficient management options to reduce disease incidence through disease suppressive microbiomes, it is important to decode the fundamental questions pertaining to plant and microbiome research.

### Tools and techniques to study rhizobacterial communities

Over 5 decades different techniques have been developed to decipher rhizobacterial traits in the context of rhizosphere competence ranging from single-gene mutagenesis techniques to -omics technologies (Barret et al., 2011). 16S rDNA-seq or shotgun DNA seq are the most commonly used techniques for the detection and quantification of microbiome composition and/or function. However, these methods have some limitations such as underrepresentation of species due to mismatches in primers (Schulz et al., 2017), low taxonomic resolution due to high DNA sequence similarity of the 16S rRNA genes (Janda and Abbott, 2007), and (typically) shorter read lengths. Complex interactions in the rhizosphere can be studied in terms of patterns in gene expression and regulation obtained through RNA sequencing of both the host and microbes (Galindo-González and Deyholos, 2016; Hayden et al., 2018). Recent investigations have garnered more interest in non-coding RNAs, operon structure and antisense RNAs to analyse the functional genomics of soil communities (Yoder-Himes et al., 2009). The investigation of sRNAs for information on post transcriptional gene regulation would provide a more extensive picture of gene regulatory networks (Prasse et al., 2017). For example, associated high quality metagenomics data (Thurber et al., 2009; Jia et al., 2020) could be linked to sRNA sequencing data to infer species associations between host and microbes at the post transcriptional regulation level. Recently, sRNA sequence data was directly used to (A) characterise host miRNA profiles and (B) conduct metagenomic analyses of the bacterial communities through homology in the bacterial sRNA databases (Mjelle et al., 2020). Although less data is available for sRNAs produced by soil microbes, similar techniques could be implemented in soil microbiome studies to complement DNA-based metagenomics data.

Currently, there are three databases available for sRNAs deposited from different bacterial species; BSRD (Li et al., 2013)**,** SRD (Sassi et al., 2015)**,** and sRNAdb (Pischimarov et al., 2012). BSRD is a comprehensive list of published bacterial sRNA sequences with annotation and expression profiles, sRD is a database for sRNAs in *Staphylococci* while sRNAdb is a database of sRNA sequences from Gram-positive bacteria. These datasets may prove useful for preliminary assessment of cross-kingdom signalling and helpful for developing hypotheses prior to initiating wet laboratory experiments.

### sRNA-mediated regulation of gene expression in the rhizosphere

**FIGURE 3:**
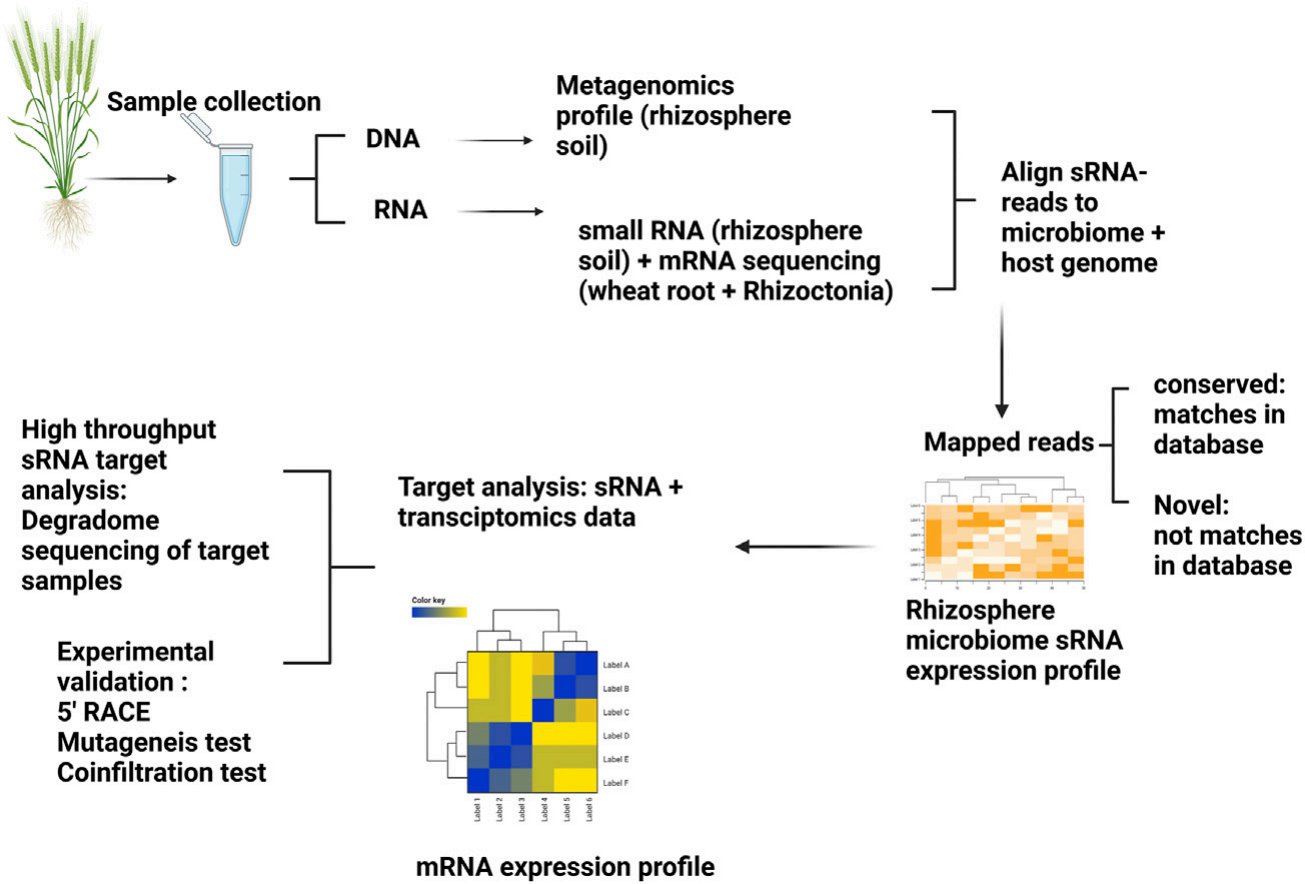
Method to study interkingdom signalling mediated by sRNAs in a wheat-rhizoctonia-rhizosphere system. At least three different types of sequencing approaches are required to test the proposed hypothesis: Plant host sRNAs that regulate the activity of bacterial and fungal genes, Fungal sRNAs that mediate the regulation of plant and bacterial mRNAs, and Bacterial sRNAs mediate the silencing of plant and fungal mRNAs. Metagenomic profiling (to characterize microbiome properties within suppressive and non-suppressive soils), small RNA sequencing (to establish the source and sRNA expression profiles in these two soils), and host + Rhizoctonia transcriptomics data (to compare the differential expressed genes identified as potential sRNA targets). In this example, we hypothesise that sRNAs originating in the rhizosphere microbiome may cross to plant tissues, thereby silencing plant genes. Therefore, we would expect sRNA expression profiles to be different in SP and NSP communities with corresponding changes to the transcriptomic profiles of wheat genes. Once the sRNA:mRNA pair predictions are made, validation of these target pairs using different molecular tools such as degradome sequencing, 5′ RACE, mutagenesis tests, and transient co-expression tests can be conducted.

Here, we present a concept to study sRNA-mediated regulation of microbiome gene expression in the rhizosphere to address the hypotheses outlined in Figure 2. Different hypotheses could be established prior to designing sequencing experiments. For example, we hypothesise there will be different sRNA profiles of microbiomes originating from suppressive and non-suppressive soils as well as contrasting host wheat root transcriptomic profiles (Figure 3). Considering that rhizosphere bacterial sRNAs are posited to silence targeted wheat genes, then the initial step would be to generate metagenomic profiles/reference-like genomes from the rhizosphere microbiome coupled with sRNA sequencing. Metagenomic and sRNA data could be used together to develop sRNA expression profiles and wheat transcriptomes utilized to generate wheat transcriptomic profiles. sRNA expression profiles could be further investigated to identify gene targets within the wheat root by utilizing a corresponding transcriptomics dataset derived from wheat grown in suppressive and non-suppressive soils. Genes identified as differentially regulated could then be mapped to the sRNA dataset to see whether sRNAs are likely regulators of those genes.

The key question is how these findings can be expanded beyond metagenomic, sRNA and metatranscriptomic datasets? Given the availability of resources, such targeted analyses could be further expanded by developing high-throughput degradome sequencing of wheat (Kumar et al., 2017) which serves to capture all degraded RNA products potentially cleaved by sRNAs. Degradome sequencing is a high-throughput method developed for transcriptome-wide detection of degraded uncapped 5′ ends of polyadenylated RNAs and has been widely used for mapping of sRNAs-mediated cleavage sites on target genes (Yu et al., 2018). Furthermore, degradome sequencing helps to offset the false positive or negative results produced by sRNA computational tools. A regulatory network of sRNA: mRNA interactions can advance the understanding of RNAi mediated gene functions within the system. As degradome typically provides hundreds to thousands of possible sRNA:mRNA interactions, the coupling of interesting functional genes and their corresponding sRNAs could be further validated experimentally in the laboratory by 5′RACE (Rapid amplification of cDNA ends) (Regmi et al., 2021), mutagenesis and/or transient co-expression tests in model plants (Wang et al., 2017a).

For example, if wheat pathogenesis related genes are silenced by *Pseudomonas* bacterial sRNAs (determined from sRNA profiling, transcriptomics dataset/degradome dataset), then it can be shown in the laboratory whether these interactions occur *in situ* b RACE experiments have been shown to be a reliable approach to show cross-kingdom RNAi in wheat (Wang et al., 2017b; Jian and Liang, 2019) but remains a low-throughput method of capturing sRNAs that mediated mRNA cleavage. Further evidence for an interaction can be gained from introducing mutations at the plant candidate cleavage site as a control (Wang et al., 2017a). Another validation technique would be assessing the interaction between sRNAs and their respective target genes by transiently expressing them in a model plant such as *Nicotiana benthamiana* (Wang et al., 2017b). These approaches require a range of microbiology, genomics, and plant molecular biology skills and as such provide an opportunity for interaction among different fields of science.

## Conclusion

The function and composition of the rhizosphere microbiome is mediated by a wide range of molecules and signals with bi-directional communication between the plant and microbiome (Mendes et al., 2013). While hosts dictate the shape of microbial communities through rhizodeposition and secretion of various molecules, microbes also modulate the environment of the plant and even reprogram the plant to their advantage (Venturi and Keel, 2016). The current understanding of cross-kingdom communication through sRNAs involves several microbe-host systems (Huang et al., 2019). However, more fundamental research is required to decode inter-kingdom communication in plant-microbiome interactions, Given the role of sRNA as key messengers that regulate different cellular process and functions, we believe the inclusion of sRNA studies in rhizosphere microbiome research will serve to shed light on some of the unanswered questions concerning disease suppression such as:

1. What are the key functions shaping the suppressive communities?
2. What is the role of the plant host in dictating and mediating suppressive communities?
3. How do different rhizosphere microorganisms communicate at intra-kingdom and inter-kingdom levels?
4. What is the role of the plant response, at a molecular level, to reduced disease impacts in suppressive *versus* non-suppressive communities?

Understanding the mechanisms shaping the microbiome community in contrasting environments, in this case suppressive and non-suppressive communities, provides a range of opportunities to modulate these communities for the benefit of agriculture through different avenues such as i) modified farming practices to encourage the development of suppressive soils by modification of the microbial communities, ii) modification of host genetics/immunity to support disease suppression, and iii) development of synthetic communities conferring stable disease suppression.
